# Association between amphetamine‐related disorders and dementia‐a nationwide cohort study in Taiwan

**DOI:** 10.1002/acn3.51113

**Published:** 2020-06-30

**Authors:** Nian‐Sheng Tzeng, Wu‐Chien Chien, Chi‐Hsiang Chung, Hsin‐An Chang, Yu‐Chen Kao, Yia‐Ping Liu

**Affiliations:** ^1^ Department of Psychiatry Tri‐Service General Hospital National Defense Medical Center Taipei Taiwan ROC; ^2^ Student Counseling Center National Defense Medical Center Taipei Taiwan ROC; ^3^ Department of Medical Research Tri‐Service General Hospital National Defense Medical Center Taipei Taiwan ROC; ^4^ School of Public Health National Defense Medical Center Taipei Taiwan, ROC; ^5^ Graduate Institute of Life Sciences National Defense Medical Center Taipei Taiwan ROC; ^6^ Taiwanese Injury Prevention and Safety Promotion Association Taipei Taiwan ROC; ^7^ Department of Psychiatry Tri‐Service General Hospital Song‐Shan Branch National Defense Medical Center Taipei Taiwan ROC; ^8^ Department of Psychiatry Chen‐Hsin General Hospital Taipei Taiwan ROC; ^9^ Laboratory of Cognitive Neuroscience Department of Physiology and Biophysics National Defense Medical Center Taipei Taiwan ROC

## Abstract

**Objective:**

We have conducted a study to clarify the association between amphetamine‐related disorders (ARD) and the risk of developing dementia.

**Methods:**

This study used a retrospective cohort design by using Taiwan’s National Health Research Institute Database. A random sample of 68,300 subjects between January 1, 2000, and December 31, 2015, was obtained, comprising of 17,075 patients with ARD, and 51,225 controls without ARD (1:3), matched for gender and age group. After adjusting for covariates, a Fine and Gray’s survival analysis (competing with mortality) was used to compare the risk of dementia during a 15‐year follow‐up period.

**Results:**

In the present study, 1,751 of 17,075 patients with ARD and 2,147 of 51,225 in the control group without ARD (883.10 vs 342.83 per 100,000 person‐years) developed dementia. ARD cohort was more likely to develop dementia (hazard ratio = 4.936 [95% CI: 4.609–5.285, *P* < 0.001). After adjusting for gender, age groups, education, monthly insured premiums, urbanization level, geographic region, comorbidities, the hazard ratio for ARD patients was 5.034 (95% CI: 4.701–5.391, *P* < 0.001). ARD has been associated with overall dementia, Alzheimer dementia, vascular dementia, and other dementia. Both the amphetamine use disorder and amphetamine‐induced psychotic disorders were associated with the risk of overall dementia, Alzheimer dementia, vascular dementia, and other dementia.

**Interpretation:**

This study shows that patients with ARD, both the amphetamine use disorder and the amphetamine‐induced psychotic disorder, may have a nearly fivefold risk of developing dementia, including Alzheimer dementia and other types of dementia.

## Introduction

Between 2011 and 2012, 130,000 people, or 4.97% of those aged 65 years and over in Taiwan had dementia,^1^ which is a heavy burden for dementia patients and their caregivers, community, and society.^2^, ^3^, ^4^, ^5^, ^6^ Injuries on the brain such as traumatic brain injury,^7^ stroke,^8^ and attention deficit hyperactivity disorder that is related traumatic brain injury,^9^, ^10^ contribute to the development of dementia. Several studies have also found that substances, such as alcohol, tobacco, and benzodiazepines, were associated with cognitive impairment, but there were no confirmed association between these substances and dementia.^11^ Other exposure to substance intoxication, such as carbon monoxide^12^, ^13^, ^14^, ^15^ or nitrogen dioxide,^12^ was also associated with the risk of dementia. The usage of central stimulants is a serious problem internationally. Amphetamine exposure has been previously reported to increase the risk of developing Parkinson’s disease in the elderly.^16^ However, the amphetamine‐related disorders (ARD) and the risk of dementia has not been investigated.

In Taiwan, most of the abused amphetamines is methamphetamine, which accounts for 40% of the illicit substance use and ranks second only to heroin.^17^ In addition, amphetamine medications, such as dextroamphetamine, are not licensed nor reimbursed by the National Health Insurance (NHI) Program in Taiwan. Furthermore, the individuals with childhood poor impulse control might tend to abuse the methamphetamine for its faster‐onset and longer‐lasting effects than the effects of amphetamine medications.^18^, ^19^ Previous studies have reported that methamphetamine has been investigated and could promote the formation of amyloid‐β42, one of the key Alzheimer’s disease‐like pathological proteins,^20^ increase the expression of the tau protein,^21^ and produce greater dementia‐related oxidative stress markers including catalase and methane dicarboxylic aldehyde.^22^ In addition, methamphetamine may modulate the functions of the immune cells and change the cytokine balance, which leads to neurotoxicity with a compromise of the blood‐brain barrier, and alterations to the brain plasticity, and eventually contributes to age‐related dysfunctions, including dementia.^23^

We hypothesize that an early exposure of amphetamine, particularly in those of amphetamine dependency, may enhance the risk of developing dementia afterwards. By employing a nationwide cohort in Taiwan, we conducted this study to investigate the association between amphetamine usage and dementia.

## Methods

### Study design and sampled participants

In this study, we used data from the National Health Insurance Research Database (NHIRD) to investigate the association between the risk of dementia in subjects with ARD cohort and the non‐ARD cohort, over a 15‐year period, from the two million Longitudinal Health Insurance Database (LHID) in Taiwan, (2000‐2015). The details of the program have been documented in previous studies.^24^, ^25^, ^26^, ^27^, ^28^, ^29^, ^30^, ^31^, ^32^, ^33^

**Figure 1:**
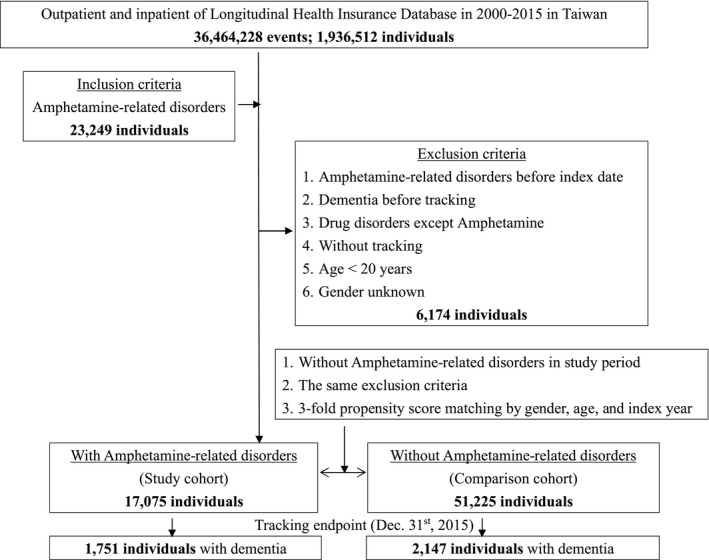
The flowchart of study sample selection.

This study was a retrospective matched‐cohort design. Patients with ARD were selected from January 1 to December 31, 2000, according to the ICD‐9‐CM codes (Table S1) as: amphetamine dependence, amphetamine abuse, and drug psychosis between January 1, 2000 and December 31, 2000, without other substance use disorders within one year before or after the diagnosis of drug psychosis, with reference from one previous study.^13^ In this study, 17,075 patients with ARD, and 51,225 in the age‐, gender‐, and index‐ year, (1:3) matched control group without ARD were enrolled, and the patients aged < 20, diagnoses of ARD and dementia before the tracking in this study were excluded (Fig. 1).

### Ethics approval and consent to participate

The Institutional Review Board of the Tri‐Service General Hospital approved this study (IRB No. 2‐107‐05‐026). The requirement for informed consent was waived by the IRB because all NHIRD data had been de‐identified.

### Material and Methods

The NHI Program was launched in Taiwan in 1995, and as of June 2009, included contracts with 97% of the medical providers with approximately 23 million beneficiaries, or more than 99% of the entire population.^34^ The NHIRD uses the International Classification of Diseases, 9th Revision, Clinical Modification (ICD‐9‐CM) codes to record diagnoses.^35^ All diagnoses of ARD were confirmed by psychiatrists according to the clinical findings, and the dementia diagnosis was made by board‐certified psychiatrists or neurologists, according to the Diagnostic and Statistical Manual of Mental Disorders, 4^th^ Edition and its Text‐revised edition.^36^, ^37^ Licensed medical records technicians also reviewed and verified the diagnostic coding before claiming the reimbursements in Taiwan’s hospitals.^38^ The NHI Administration randomly reviews the records of ambulatory care visits and inpatient claims periodically to verify the accuracy of the diagnose.^39^ Therefore, it is suitable using the NHIRD to study the association between ARD and dementia.

The covariates included gender, age (20–49, 50–64, ≧65), education (<12 years, ≧12 years), geographical area of residence (north, center, south, and east of Taiwan), urbanization level of residence (levels 1 to 4), levels of hospitals as medical centers, regional and local hospitals, and insurance premium (in New Taiwan Dollars [NT$]; <18,000, 18,000–34,999, ≥35,000). The urbanization level of residence was defined according to the population and various indicators of the level of development. Level 1 was defined as a population of >1,250,000, and a specific designation as political, economic, cultural, and metropolitan development. Level 2 was defined as a population between 500,000 and 1,249,999, and as playing an important role in the political system, economy, and culture. Urbanization levels 3 and 4 were defined as a population between 149,999 and 499,999, and <149,999 respectively.^12^ The Charlson Comorbidity Index (CCI) was also used to categorize the comorbidities using the ICD‐9‐CM codes, scores each comorbidity category,^40^, ^41^, ^42^ and combines all the scores to calculate a single comorbidity score. A score of zero indicates that no comorbidities were found, and higher scores indicate higher comorbidity burdens.^43^

All the study participants were followed from the index date until the onset of dementia including Alzheimer dementia, vascular dementia (VaD), and other degenerative dementia, according to the ICD‐9‐CM codes (Table S1), withdrawal from the NHI program, or the end of 2015.

### Statistical analysis

All analyses were performed using the SPSS software version 22 (SPSS Inc., Chicago, Illinois, USA). χ^2^ and t tests were used to evaluate the distributions of the categorical and continuous variables respectively. In addition, the Fisher exact test for categorical variables was used to statistically examine the differences between the two cohorts, while the sample size was < 5. Fine and Gray’s survival analysis was used to determine the risk of dementia, and the results were presented as a hazard ratio (HR) with a 95% confidence interval (CI). The Fine and Gray’s model benefits from the use of the inclusion of the actual mortality data to study the usage of methamphetamine as a risk factor for dementia.^44^, ^45^ The difference in the risk of dementia, between the study and control groups, was estimated using the Kaplan–Meier method with the log‐rank test. A 2‐tailed *P* value < 0.05 was considered to indicate statistical significance.

## Results

### Baseline characteristics

Table 1 depicts that the ARD cohort tended to have a lower education level, insured premiums < 18,000 and ≧35,000, CCI score of zero, living in northern and southern, residence of urbanization levels 1 and 2, and seeking medical help in the regional hospitals. There were no significant differences between ARD and non‐ARD cohorts in other covariates.

**Table 1:** Characteristics of study subjects at the baseline

Amphetamine‐related disorders	With		Without		*P*
Variables	*n*	%	*n*	%	
Total	17,075	25.00	51,225	75.00	
Gender					0.999
Male	11,992	70.23	35,976	70.23	
Female	5,083	29.77	15,249	29.77	
Age (years)	43.85 ± 18.40		44.12 ± 16.98		0.078
Age group (years)					0.999
20–49	12,185	71.36	36,555	71.36	
50–64	1,750	10.25	5,250	10.25	
≧65	3,140	18.39	9,420	18.39	
Education (years)					<0.001
0–11	11,840	69.34	17,771	34.69	
≧12	5,235	30.66	33,454	65.31	
Insured premium (NT$)					<0.001
<18,000	16,280	95.34	46,787	91.34	
18,000‐34,999	234	1.37	3,312	6.47	
≧35,000	561	3.29	1,126	2.20	
CCI_R group					<0.001
0	13,137	76.94	37,950	74.08	
1	2,185	12.80	6,890	13.45	
2	781	4.57	2,508	4.90	
3	443	2.59	1,958	3.82	
≧4	529	3.10	1,919	3.75	
Location					<0.001
Northern Taiwan	7,379	43.22	20,708	40.43	
Middle Taiwan	4,075	23.87	14,135	27.59	
Southern Taiwan	4,612	27.01	13,126	25.62	
Eastern Taiwan	974	5.70	3,034	5.92	
Outlets islands	35	0.20	222	0.43	
Urbanization level					<0.001
1 (The highest)	5,999	35.13	17,617	34.39	
2	7,317	42.85	21,063	41.12	
3	1,318	7.72	4,585	8.95	
4 (The lowest)	2,441	14.30	7,960	15.54	
Level of care					<0.001
Medical center	5,495	32.18	15,064	29.41	
Regional hospital	9,081	53.18	15,638	30.53	
Local hospital	2,499	14.64	20,523	40.06	

*P,* Chi‐square/ Fisher exact test on category variables and t‐test on continue variables; NT$, New Taiwan Dollars; CCI_R, Charlson Comorbidity Index, dementia removed.

### Kaplan–Meier model for the cumulative risk of dementia

**Figure 2:**
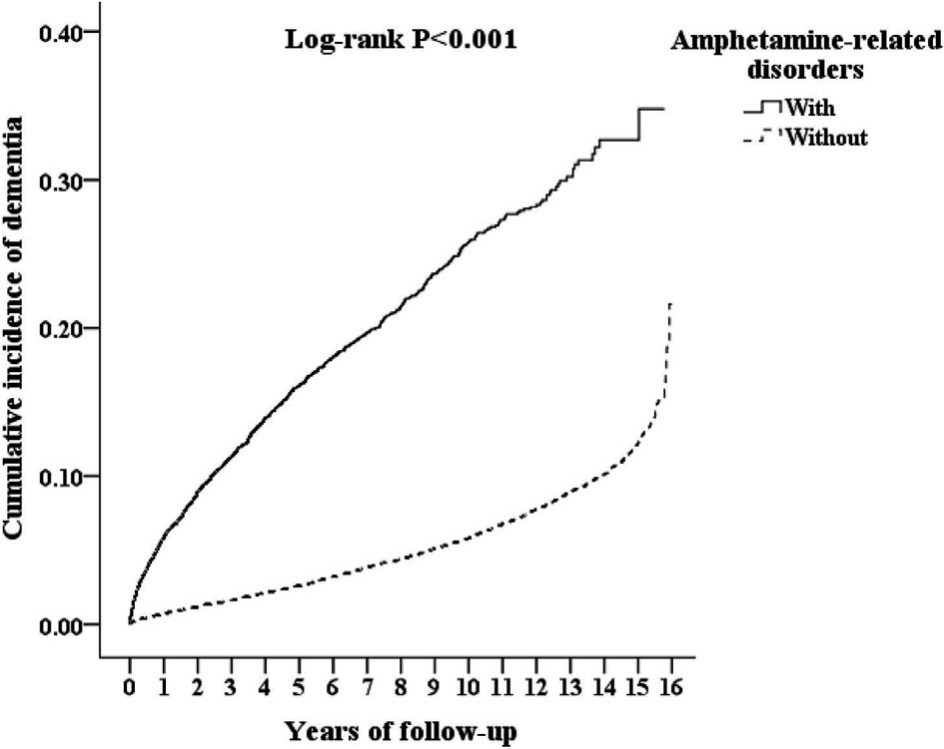
Kaplan–Meier for cumulative incidence of dementia aged 20 and over stratified by Amphetamine‐related disorders with log‐rank test.

In the present study, 1,751 of 17,075 patients with ARD (10.25%), and 2,147 of 51,225 (4.19%) in the control group without ARD (883.10 vs. 342.83 per 100,000 person‐years) developed dementia. The Kaplan–Meier analysis revealed that the ARD cohort had a significantly higher 15‐year dementia cumulative incidence rate than the controls. (log‐rank, *P* < 0.001, Fig. 2). Figure S1 has shown the survival data as the dementia‐free survival for patients with and without amphetamine‐related disorders during the 15‐year follow‐up period in Taiwan (log‐rank, *P* < 0.001).

### Hazard ratios analysis of dementia in the ARD cohort

Table 2 shows that the ARD cohort was more likely to develop dementia (hazard ratio = 4.936 [95% CI:4.609–5.285, *P* < 0.001). After adjusting for gender, age groups, education, monthly insured premiums, urbanization level, geographic region, comorbidities, the hazard ratio for ARD patients was 5.034 (95% CI: 4.701–5.391, *P* < 0.001), when compared to the non‐ARD control cohort. In other words, the ARD cohort has a fivefold risk of dementia, when compared to the non‐ARD cohort. Furthermore, male patients with ARD were associated with the risk of dementia (adjusted HR = 1.315, 95% CI: 1.227–1.410, *P* < 0.001), in comparison to the female patients. The ARD cohort with those aged ≧65, educational years of 0‐11, scores of CCI as 1, 3, and 4, and urbanization levels of 1, 2, and 3, was associated with the increased risk of dementia. On the other hand, the ARD, with those aged 50‐64, insurance premiums of NT$ 18,000‐34,999, and seeking medical care from the medical centers and regional hospitals, was associated with the decreased risk of dementia.

**Table 2:** Factors of dementia by using Fine and Gray’s competing risk model

	No competing risk in the model				Competing risk in the model			
Variables	Adjusted HR	95% CI	95% CI	*P*	Adjusted HR	95% CI	95% CI	*P*
Amphetamine‐related disorders								
Without	Reference				Reference			
With	4.936	4.609	5.285	<0.001	5.034	4.701	5.391	<0.001
Gender								
Male	1.275	1.189	1.367	<0.001	1.315	1.227	1.410	<0.001
Female	Reference				Reference			
Age group (years)								
20–49	Reference				Reference			
50–64	0.753	0.675	0.840	<0.001	0.775	0.695	0.865	<0.001
≧65	3.327	3.081	3.593	<0.001	3.836	3.551	4.145	<0.001
Education (years)								
0–11	1.407	1.057	1.972	0.011	1.451	1.071	2.005	0.001
≧12	Reference				Reference			
Insured premium (NT$)								
<18,000	Reference				Reference			
18,000–34,999	0.640	0.465	0.881	0.006	0.641	0.466	0.883	0.006
≧35,000	0.425	0.160	1.135	0.088	0.433	0.162	1.155	0.095
CCI_R group								
0	Reference				Reference			
1	1.051	0.971	1.137	0.218	1.084	1.002	1.173	0.045
2	0.973	0.872	1.086	0.628	1.053	0.943	1.175	0.359
3	0.632	0.540	0.738	<0.001	0.729	0.623	0.852	<0.001
≧4	0.359	0.303	0.426	<0.001	0.487	0.410	0.578	<0.001
Urbanization level								
1 (The highest)	0.779	0.705	0.860	<0.001	0.818	0.740	0.904	<0.001
2	0.797	0.731	0.868	<0.001	0.816	0.749	0.889	<0.001
3	0.697	0.612	0.794	<0.001	0.692	0.608	0.788	<0.001
4 (The lowest)	Reference				Reference			
Level of care								
Medical center	0.784	0.712	0.864	<0.001	0.756	0.686	0.833	<0.001
Regional hospital	0.803	0.743	0.867	<0.001	0.780	0.722	0.842	<0.001
Local hospital	Reference				Reference			

HR, hazard ratio; CI, confidence interval; Adjusted HR, Adjusted variables listed in the Table 1; NT$, New Taiwan Dollars; CCI_R, Charlson Comorbidity Index, dementia removed.

### Subgroup analysis in the association between ARD and dementia

The ARD cohort was associated with the increased risk of dementia, in each demographic factor, covariates, scores of CCI, urbanization levels, and the care from the either medical centers, regional hospitals, and local hospitals (Table 3).

**Table 3:** Factors of dementia stratified by variables listed in the table by using Fine & Gray's competing risk model

Amphetamine‐related disorders	With			Without			Competing risk in the model			
Stratified		PYs	Rate (per 10^5^ PYs)	Events	PYs	Rate (per 10^5^ PYs)	Adjusted HR	95% CI	95% CI	*P*
Overall	1,751	198,278.28	883.10	2,147	626,249.70	342.83	5.034	4.701	5.391	<0.001
Gender										
Male	1,254	138,316.11	906.62	1,453	441,082.28	329.42	5.379	5.023	5.760	<0.001
Female	497	59,962.17	828.86	694	185,167.42	374.80	4.322	4.036	4.628	<0.001
Age group (years)										
20–49	931	126,751.31	734.51	432	241,275.04	179.05	8.017	7.487	8.586	<0.001
50–64	162	28,046.02	577.62	303	201,048.23	150.71	7.490	6.995	8.021	<0.001
≧65	658	43,480.95	1,513.31	1,412	183,926.43	767.70	3.852	3.597	4.126	<0.001
Education (years)										
0–11	865	75,012.46	1,153.14	1,011	232,121.02	435.55	5.174	4.832	5.541	<0.001
≧12	886	79,784.87	1,110.49	1,136	210,202.25	540.43	4.016	3.750	4.300	<0.001
Insured premium (NT$)										
<18,000	1,732	195,339.81	886.66	2,124	612,609.23	346.71	4.998	4.667	5.352	<0.001
18,000–34,999	18	2,780.93	647.27	20	10,324.80	193.71	6.530	6.098	6.993	<0.001
≧35,000	1	157.54	634.77	3	3,315.67	90.48	13.710	12.803	14.683	<0.001
CCI_R group										
0	1,221	132,500.97	921.50	916	342,837.75	267.18	6.740	6.294	7.218	<0.001
1	339	34,307.78	988.11	687	128,549.44	534.42	3.613	3.374	3.870	<0.001
2	112	12,093.61	926.11	300	52,902.59	567.08	3.192	2.980	3.418	<0.001
3	42	7,563.04	555.33	137	39,535.27	346.53	3.132	2.925	3.354	<0.001
≧4	37	11,812.89	313.22	107	62,424.66	171.41	3.571	3.335	3.824	<0.001
Urbanization level										
1 (The highest)	438	57,312.28	764.23	581	187,974.99	309.08	4.832	4.512	5.175	<0.001
2	730	86,591.99	843.03	926	272,868.75	339.36	4.855	4.534	5.199	<0.001
3	152	18,694.55	813.07	152	55,027.04	276.23	5.752	5.372	6.160	<0.001
4 (The lowest)	431	35,679.46	1,207.98	488	110,378.91	442.11	5.340	4.986	5.718	<0.001
Level of care										
Hospital center	351	52,553.35	667.89	606	204,099.71	296.91	4.396	4.105	4.708	<0.001
Regional hospital	817	98,589.28	828.69	926	283,123.01	327.07	4.952	4.624	5.303	<0.001
Local hospital	583	47,135.65	1,236.86	615	139,026.98	442.36	5.464	5.103	5.852	<0.001

PYs, Person‐years; Adjusted HR, Adjusted Hazard ratio: Adjusted for the variables listed in Table 1.; CI, confidence interval; NT$, New Taiwan Dollars.

### Risk of the types of dementia in the ARD cohort and the sensitivity analysis

Table 4 depicts that the ARD cohort was associated with overall dementia, AD, VaD, and other dementia in the overall follow‐up period. In addition, the ARD cohort was associated with overall dementia, AD, VaD, and other dementia, after the exclusion of dementia diagnosis of the first two years after the diagnosis of ARD. The ARD cohort was associated with overall dementia, AD, and other dementia, but not VaD, after the exclusion of the dementia diagnosis of the first five years after the diagnosis of ARD.

**Table 4:** Factors of dementia subgroup and sensitivity test by using Fine and Gray’s competing risk model

	Amphetamine‐related disorders	No competing risk in the model				Competing risk in the model			
Sensitivity test	Dementia subgroup	Adjusted HR	95% CI	95% CI	*P*	Adjusted HR	95% CI	95% CI	*P*
Overall	Overall dementia	4.936	4.609	5.285	<0.001	5.034	4.701	5.391	<0.001
	AD	3.806	3.554	4.075	<0.001	3.882	3.621	4.157	<0.001
	VaD	2.460	2.297	2.634	<0.001	2.509	2.340	2.687	<0.001
	Other dementia	5.196	4.852	5.564	<0.001	5.301	4.949	5.678	<0.001
First 2 years excluded	Overall dementia	2.862	2.671	3.065	<0.001	2.918	2.725	3.128	<0.001
	AD	2.227	2.080	2.389	<0.001	2.271	2.122	2.439	<0.001
	VaD	1.580	1.476	1.692	<0.001	1.613	1.502	1.729	<0.001
	Other dementia	2.999	2.798	3.213	<0.001	3.055	2.851	3.270	<0.001
First 5 years excluded	Overall dementia	1.922	1.725	2.085	<0.001	1.960	1.833	2.099	<0.001
	AD	1.313	1.021	1.534	0.022	1.339	1.101	1.443	0.004
	VaD	1.100	0.862	1.259	0.267	1.128	0.953	1.080	0.136
	Other dementia	2.205	1.890	2.169	<0.001	2.065	1.928	1.212	<0.001

PYs, Person‐years; Adjusted HR, Adjusted Hazard ratio, Adjusted for the variables listed in Table 3.; CI, confidence interval; AD, Alzheimer dementia; VaD, vascular dementia.

In addition, both the amphetamine use disorder and amphetamine‐induced psychotic disorder were associated with the risk of overall dementia, AD, VaD, and other dementia (Table 5).

**Table 5:** Factors of dementia subgroup stratified by amphetamine‐related disorders by using Fine and Gray’s competing risk model

Amphetamine‐related disorders	Dementia types	Events	Adjusted HR	95% CI	95% CI	*P*
Amphetamine use disorder (reference: without)	Overall	200	3.189	2.971	3.419	<0.001
Amphetamine use disorder (reference: without)	AD	9	3.172	2.955	3.401	<0.001
Amphetamine use disorder (reference: without)	VaD	10	2.207	2.061	2.375	<0.001
Amphetamine use disorder (reference: without)	Other dementia	181	3.259	3.029	3.514	<0.001
Amphetamine‐induced psychotic disorder (reference: without)	Overall	1,551	5.440	5.072	5.821	<0.001
Amphetamine‐induced psychotic disorder (reference: without)	AD	52	4.036	3.765	4.333	<0.001
Amphetamine‐induced psychotic disorder (reference: without)	VaD	53	2.574	2.404	2.779	<0.001
Amphetamine‐induced psychotic disorder (reference: without)	Other dementia	1,446	5.740	5.352	6.180	<0.001

PYs, Person‐years; Adjusted HR, Adjusted Hazard ratio, Adjusted for the variables listed in Table 1.; CI, confidence interval.

## Discussion

### Association between ARD cohort and the risk of dementia

In this study, we found that in the 15‐year follow‐up, the ARD cohort was associated with a higher risk of developing dementia. The log rank of the Fine and Gray’s competing risks regression model was significant (*P* < 0.001). The crude HR of the subject group was 4.936 (95% CI: 4.609–5.285, *P* < 0.001), and the adjusted HR was 5.034 (95% CI: 4.701–5.391, *P* < 0.001). The ARD cohort has a fivefold risk of dementia, when compared to the non‐ARD cohort. A subgroup analysis also found that the patients with ARD were associated with the risk of dementia, in all the demographic factors, covariates, scores of CCI, urbanization levels, and the care from the either medical centers, regional and local hospitals, in comparison to the controls without ARDs. We have also conducted two sensitivity analyses to evaluate the influences from protopathic bias. Even though the patients with a diagnosis of dementia within the first two years and five years were excluded, the ARD cohort were still associated with an increased risk of overall dementia and each types of dementia, except for the association between ARD and VaD after the exclusion of the first five years of dementia. To the best of our knowledge, this is the first nationwide, population‐based cohort study that has focused on the association between the ARD cohort and the risk of dementia.

In the present study, the cumulative incidence of dementia within 15 years was 5.9% in all the enrolled subjects, and 4.2% in the controls without ARD, which is compatible to the prevalence of dementia as 2–5%.^46^, ^47^, ^48^

In addition, the 1‐year (between January 1, and December 31, 2000) prevalence rate of ARD was 0.88%, which was just slightly lower than, but close to, that in an epidemiological study for the male patients with methamphetamine use disorder in one county (Taoyuan), as 1.24%, in 2002.^49^ However, given the fact that it is difficult to estimate the actual prevalence of illicit drug use disorders, a population‐based study would be a reasonable estimation of the ARD in Taiwan.

### Comparison to previous literature

Several previous studies have depicted the association between the medical usage of several drugs and the risk of dementia, such as high doses of opioids,^50^ proton pump inhibitors,^51^, ^52^, ^53^ benzodiazepines,^54^ disease‐modifying antirheumatic drugs, anticholinergics,^55^ and even some types of antidepressants.^56^ However, few reports have denoted the association of substance use disorders and the risk of dementia, with the exception of the alcohol use disorder.^5^, ^57^, ^58^, ^59^ In addition, the abuse of amphetamines was associated with the risk of Parkinson’s disease, another neurodegenerative disease, in previous studies, including amphetamine and methamphetamine.^60^, ^61^, ^62^ Nonetheless, this is the first on the issues of the association between ARD and risk of dementia. In this study, both the amphetamine use disorders and amphetamine‐induced psychotic disorders were associated with the risk of overall dementia, AD, VaD, and other dementia.

### Potential mechanisms of the association between ARD and risk of dementia

The cardinal finding of this study is that we confirm an earlier exposure of amphetamine in Taiwan which led to a higher risk of developing dementia. The potential mechanisms can be twofold. First, as we mentioned earlier, through its central impacts, amphetamine may influence those neurosubstrates associated to the underlying mechanism of dementia, namely, beta‐amyloid cascade, tau protein, oxidative stress, and neural inflammation,^63^, ^64^ and of which are a time‐consuming process. For example, microglial activation may well be associated to the chronic inflammatory process,^65^ and the chronic inflammation condition may contribute to the pathophysiology of AD,^66^ and vascular dementia.^67^ Second, the image positron emission tomography (PET) study revealed that excessive usage of hedonic substances results in a down‐regulation of the D2 receptors, thus, the addicts need to take “more” and the abstinent users have difficulty experiencing pleasure with the natural reinforcers of life.^68^ This appears a possibility of a downward change of brain function following the usage of a cognitive enhancer, which may be interpreted by the opponent process theory,^69^, ^70^ which was first introduced by Solomon and Corbit (1974, 1980), and is usually employed in the interpretation of drug dependence of central stimulants, which suggests that following a positive hedonic response (upward change above the baseline, a‐process), homeostatic changes in the brain circuits may function to dampen this positive response (downward change below the baseline, b‐process).^69^ in terms of drug addiction of the central stimulants, and the compulsive drug consumption can be viewed as a negative reinforcing course to compensate the b‐process. Usually, the a‐process occurs rapidly after the usage of the drug. As to the b‐process, it goes with a slow onset, and in its process, the body exerts itself to achieve homeostasis through change, in which the accumulating brain damage resulting from this chronic aberration, as time goes by, is referred to as allostatic load.^71^, ^72^

Therefore, if we widen the time scale to a life‐long period and consider the earlier usage of amphetamines as an exposure of the cognitive enhancer which causes upward change of cognition in the beginning,^73^ it is possible that the *b‐*process, on the other hand, reflects its serious allostatic load on a downward change of cognition from the baseline in a quite procrastination manner. In point of fact, for those experiencing earlier amphetamine exposure the cognitive deterioration can be highly regarded as a specific form of compensatory change of the brain function following the exposure of the cognition enhancer, such as amphetamine, thus rendering a high risk of development dementia.

It is interesting to note that in the present study, the ARD cohort was associated with overall dementia, AD, and other dementia, but not VaD. The underlying mechanism could be complicated, however, as it is possibly relevant to the observation that the amphetamine‐associated stroke often occurs when closely following the drug exposure rather than a postponement until elderly,^16^ yet dementia diagnosis of the first five years after the diagnosis of ARD, however, was excluded in this study.

Additionally, in the present study, the ARD cohort with monthly insured premiums of ≧ 35,000, in the residence of higher urbanization, and those that received their medical care from the medical centers and regional hospitals, tended to have a lower risk of dementia. Furthermore, the ARD cohort with a lower educational level was associated with a higher risk of dementia. The socioeconomic levels might also play an important role in the development of dementia. The underlying mechanisms between the association of ARD and the risk of dementia need more studies.

### Age and gender effects in the risk of dementia

Several previous studies have reported that aging itself is a risk factor of dementia development.^46^, ^47^, ^48^, ^74^ ARD patients aged 50–64 were associated with a higher risk of dementia, and the ARD cohort aged ≧65 was associated with a lower risk of dementia in this study, in comparison to those aged 20–49. The reason for this discrepancy might be related to the previous findings that most of the users of amphetamine were middle aged,^49^ and a 15‐year period of follow‐up might be enough for these patients to develop the progressive neurodegenerative process to develop dementia. We hypothesize that the effects of amphetamine exposure are life‐stage dependent. And the earlier exposure of amphetamine is the more liable to develop a dementia in later life. When the ARD cohort is aged ≧65, the risk reduces. The interpretation is also along with our hypothesis of the opponent process theory that an earlier exposure to amphetamine, the more readiness for the body to develop a homeostatic change in brain circuits to dampen the positive response.

In addition, male ARD patients were associated with the increased risk of dementia, in comparison to the female patients. The gender effects varied in the risks for different types of dementia as follows.^75^, ^76^ Further studies may well be needed to examine the association among male patients, anticholinergic usage, and the risk of dementia.

### Childhood self‐control, cognitive ability, ARD, and the risk of dementia

In the present study, the cases with other addictions were excluded in both ARD and control cohorts. In addition, when excluding cases developed dementia within first two and first five years, the risks of dementia were significantly decreased, in the sensitivity analysis. Thus, it is possible that drug addiction itself, not just ARD, was associated with dementia. Therefore, a common earlier life event or factor may have contributed to both ARD and dementia. One previous study, in Dunedin, New Zealand, found that the childhood self‐control could predict the physical health, substance dependence, personal finances, and criminal offending outcomes. Lower self‐control had poorer outcomes, including increased risk of substance use disorder.^77^ In addition, there might be an indirect connection between the childhood self‐control and dementia, since several studies have found the association between ADHD and risk of dementia^9^, ^10^ and the poor childhood self‐control is one of the core problems of ADHD.^78^ Besides, the individuals with childhood poor impulse control might tend to abuse the methamphetamine for its fast‐onset and long‐lasting effects.^18^, ^19^ Therefore, further studies are needed to investigate the association among ARD, childhood self‐control, and the risk of dementia.

One case–control study in Scotland, the United Kingdom, has found that lower premorbid cognitive intelligence quotient (IQ) in childhood increases VaD development in later life.^79^ This finding also indicates that VaD could occur much earlier than other dementia. The earlier occurrence of VaD might agree with the finding that the ARD cohort was associated with overall dementia, AD, and other dementia, but not VaD, after the exclusion of the dementia diagnosis of the first five years after the diagnosis of ARD, in the sensitivity analysis. Besides, the reports about the association between childhood IQ and risk of adult illicit substance abuse varied. Two civilian cohort studies found that high childhood IQ may increase the risk of illegal drug use in adolescence, adulthood, and middle age.^80^, ^81^ However, one study in the United States Army found that male veterans with high childhood IQ was associated with less likely to be habitual users of cannabis, cocaine, heroin, amphetamines, barbiturates, and lysergic acid diethylamide (LSD).^82^ Therefore, further studies are needed to clarify the link among childhood IQ, VaD, and illicit substance use disorders, including ARD.

### Limitations

There were several limitations in the present study. First, our study is retrospective using the ICD‐9‐CM codes, therefore, the lack of detailed records of genetic, nutritional, habitual factors, smoking, and body mass index that were not included in such a claims database study. Second, this national review insurance database cannot provide detailed information, including the severity, stage, and the care‐giver burden of the patients with dementia. Furthermore, Alzheimer dementia is the most common cause of dementia (40–60% in all dementias), followed by vascular dementia (20–30% in all dementias), and mixed or other dementias (7–15%) from previous studies in Taiwan,^46^, ^47^ but most of the dementia in our study were other types of dementia. The possibility is that clinicians might put these types of dementia with a nature of progressive and gradual decline and no evidence of previous cerebrovascular events, into this category instead of AD.

## Conclusion

This study shows that patients with ARD, both amphetamine use disorder and amphetamine‐induced psychotic disorder, may have a nearly fivefold risk of developing dementia in comparison to the non‐ARD cohort. This result could serve as a reminder for clinicians who are in charge of the care of patients with ARD.

## Conflict of Interest

None.

## Supporting information


**Figure S1.** Dementia‐free survival for patients with and without amphetamine‐related disorders during the 15‐year follow‐up period in Taiwan.

**Table S1.** ICD‐9‐CM codes.
