# Supplementary figures and images for: Association between amphetamine‐related disorders and dementia‐a nationwide cohort study in Taiwan
Source: Ann Clin Transl Neurol. 2020 Jun 30;7(8):1284–95. doi: 10.1002/acn3.51113 (PMC7448166; doi:10.1002/acn3.51113)

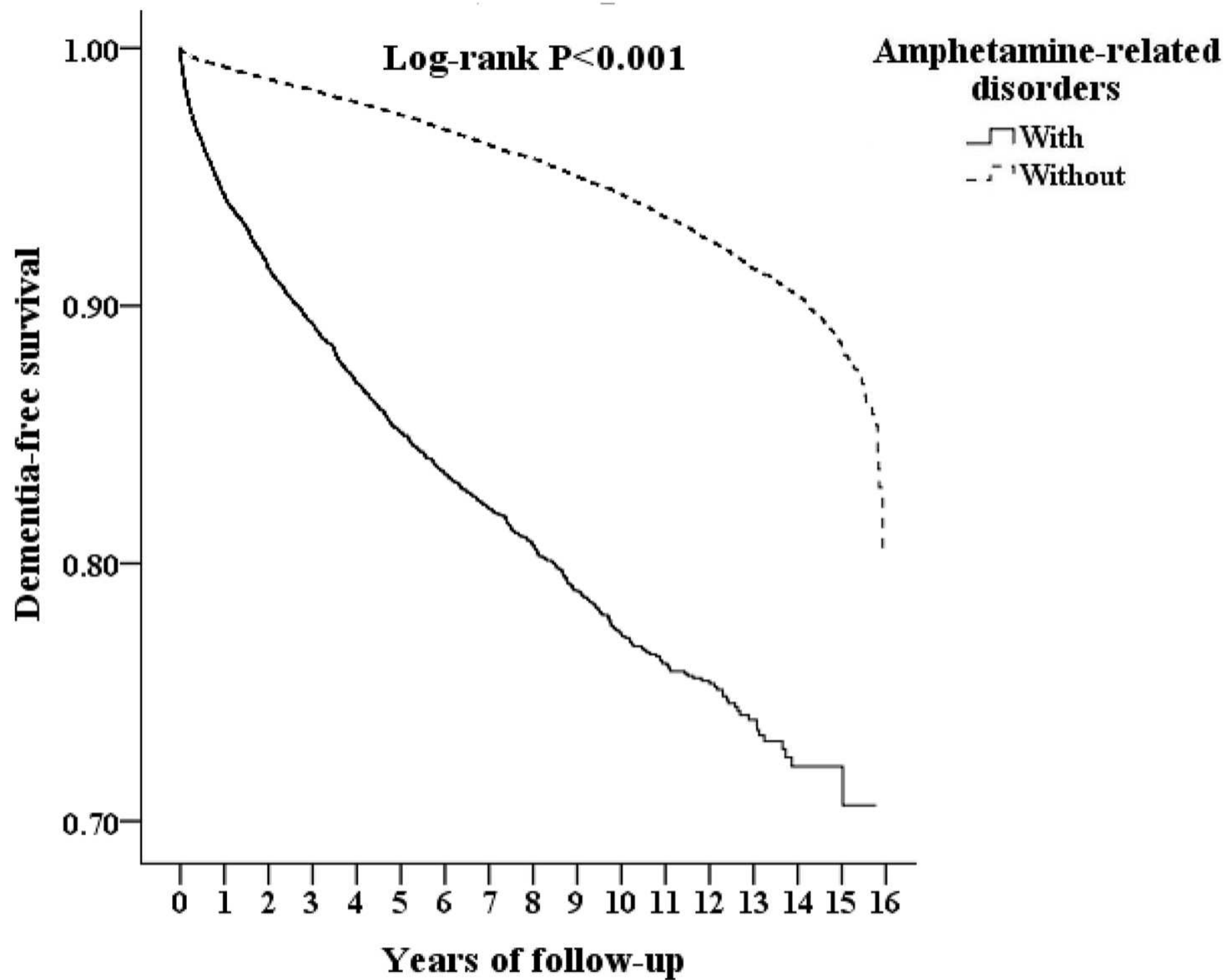

Supplement: Supplementary file 1 — Figure S1. Dementia‐free survival for patients with and without amphetamine‐related disorders during the 15‐year follow‐up period in Taiwan. [file ACN3-7-1284-s001.pdf]
