# Supplementary material for: Association between amphetamine‐related disorders and dementia‐a nationwide cohort study in Taiwan
Source: Ann Clin Transl Neurol. 2020 Jun 30;7(8):1284–95. doi: 10.1002/acn3.51113 (PMC7448166; doi:10.1002/acn3.51113)
Supplement: Supplementary file 2 — Table S1. ICD‐9‐CM codes. [file ACN3-7-1284-s002.doc]

| **Table S1. ICD-9-CM codes** | |
| --- | --- |
|  | **ICD-9-CM codes** |
| Amphetamine-related disorders |  |
| Amphetamine dependence | 304.4 |
| Amphetamine abuse | 350.7 |
| Amphetamine psychosis | 292 |
| Drug disorders | 304.0-304.3, 304.5-304.9, 350.0-350.6, 350.8-350.9 |
| Overall dementia | 290.0, 290.10-290.13, 290.20-290.21, 290.3. 290.40-290.43, 290.8-290.9, 331.0 |
| Alzheimer's disease | 331.0 |
| Vascular dementia | 290.4 |
| Other degenerative dementia | 290.x except 290.4 |

**ICD-9-CM = International Classification of Disease, 9^th^ edition, Clinical Manual**
